# From crystal structure of α-conotoxin GIC in complex with *Ac*-AChBP to molecular determinants of its high selectivity for α3β2 nAChR

**DOI:** 10.1038/srep22349

**Published:** 2016-03-01

**Authors:** Bo Lin, Manyu Xu, Xiaopeng Zhu, Yong Wu, Xi Liu, Dongting Zhangsun, Yuanyan Hu, Shi-Hua Xiang, Igor E. Kasheverov, Victor I. Tsetlin, Xinquan Wang, Sulan Luo

**Affiliations:** 1 Key Laboratory of Tropical Biological Resources, Ministry of Education, Key Lab for Marine Drugs of Haikou, Hainan University, Haikou Hainan 570228, P. R. China; 2 Ministry of Education Key Laboratory of Protein Science, Beijing Advanced Innovation Center for Structural Biology, Collaborative Innovation Center for Biotherapy, School of Life Sciences, Tsinghua University, Beijing 100084, P. R. China; 3 Shemyakin-Ovchinnikov Institute of Bioorganic Chemistry, Russian Academy of Sciences, Miklukho-Maklaya Street, 16/10 Moscow 117997, Russia; 4 Nebraska Centre for Virology, School of Veterinary Medicine and Biological Sciences, University of Nebraska-Lincoln, Lincoln, NE 68583, USA

## Abstract

Acetylcholine binding proteins (AChBPs) are unique spatial homologs of the ligand-binding domains of nicotinic acetylcholine receptors (nAChRs), and they reproduce some pharmacological properties of nAChRs. X-ray crystal structures of AСhBP in complex with α-conotoxins provide important insights into the interactions of α-conotoxins with distinct nAChR subtypes. Although considerable efforts have been made to understand why α-conotoxin GIC is strongly selective for α3β2 nAChR, this question has not yet been solved. Here we present the structure of α-conotoxin GIC in complex with *Aplysia californica* AChBP (*Ac*-AChBP) at a resolution of 2.1 Å. Based on this co-crystal structure complemented with molecular docking data, we suggest the key residues of GIC in determining its high affinity and selectivity for human α3β2 vs α3β4 nAChRs. These suggestions were checked by radioligand and electrophysiology experiments, which confirmed the functional role of detected contacts for GIC interactions with *Ac*-AChBP and α3β2 nAChR subtypes. While GIC elements responsible for its high affinity binding with *Ac*-AChBP and α3β2 nAChR were identified, our study also showed the limitations of computer modelling in extending the data from the X-ray structures of the AChBP complexes to all nAChR subtypes.

The discovery and crystallization of the acetylcholine-binding protein (AChBP) from a fresh-water mollusc *Lymnaea stagnalis*^1^,^2^ was a great step in understanding the structure and function of both nicotinic acetylcholine receptors (nAChR) and other ligand-gated ion channels belonging to the family of Cys-loop receptors. The first X-ray structure^2^, based on the disposition of the bound buffer molecule, provided the first ideas about the localization of the binding sites at the interface between the AChBP subunits and also between those of true nAChRs. It was confirmed by the subsequent X-ray structure of the AChBP complex with nicotine^3^, a classical agonist for most of the nAChRs subtypes. Interestingly, this structure revealed that nicotine attachment is accompanied by considerable movement of the AChBP loop C from the periphery to the central axis, and finally, nicotine appears to be embraced by this loop. In contrast, one year later, the X-ray structures for AChBP complexes with antagonists such as α-cobratoxin^4^ or α-conotoxin PnIA (A10L, D14K)^5^ demonstrated that the loop C is shifted to the periphery by more than 10 Å. In fact, the first X-ray structures for AChBP itself and its complexes with nicotine and α-cobratoxin have been established for *Lymnaea stagnalis* protein (*Ls*-AChBP), while the majority of the presently known AChBP complexes were solved with AChBP from the sea-water mollusc *Aplysia californica* (*Ac*-AChBP) (see recent reviews^6^,^7^,^8^,^9^,^10^). These two proteins have remarkable differences in their affinity both to α-neurotoxins and different α-conotoxins and this is one of the factors that makes a step from the high-resolution X-ray structure of the AChBP complexes to the expected structures of the distinct nAChRs binding the same ligands more difficult. Other complications are due to the limitations of computer modelling methods at the stage of going from the coordinates of the AChBP complexes to the precise organization of the analysed particular nAChR subtype.

Our work focused on the *Ac*-AChBP interaction with α-conotoxins. The advantage of α-conotoxins is that, among other antagonists, they are the most selective tools that allow distinguishing distinct subtypes of nAChRs^6^,^7^,^8^,^9^,^10^. In spite of the availability of the X-ray structures of *Ac*-AChBPs complexes with different α-conotoxins^5^,^11^,^12^,^13^, further work is required to understand the specificity of interactions of α-conotoxins with distinct nAChR subtypes with complete details. Here we analysed the α-conotoxin GIC bound to the neuronal α3β2 nAChR. These receptors are present in the human brain, are involved in functions such as cognition and, among other neuronal nAChRs, are considered to be promising drug targets (see reviews^14^,^15^,^16^). α-Conotoxin GIC from *Conus geographus* venom is an extremely interesting peptide that potently and selectively blocks neuronal α3β2 nAChRs at very low concentrations (IC_50_ 1.1 nM). This high selectivity for the human neuronal α3β2 receptor makes it one of the most attractive cholinergic ligands found in recent years. Although many efforts have been made to understand the high selectivity of GIC for the human α3β2 nAChR^17^,^18^,^19^, the answer is still not clear. In this report, we present the co-crystal structure at a resolution of 2.1 Å of the α-conotoxin GIC in complex with *Ac*-AChBP as a further step to shed light on the selectivity mechanisms in nAChRs research.

## Results

### Overall structure of the complex

The *Ac-*AChBP expressed in insect cells was purified and co-crystallized with synthesized α-conotoxin GIC using the vapour diffusion sitting drop method. The crystals of the protein-peptide complex belong to the *P*2_1_2_1_2_1_ space group with cell dimensions of a = 78.6 Å, b = 84.9 Å, and c = 208.6 Å (Supplementary Table 1). The crystal structure was determined by molecular replacement and refined to a resolution of 2.1 Å (Supplementary Table 1).

**Figure 1:**
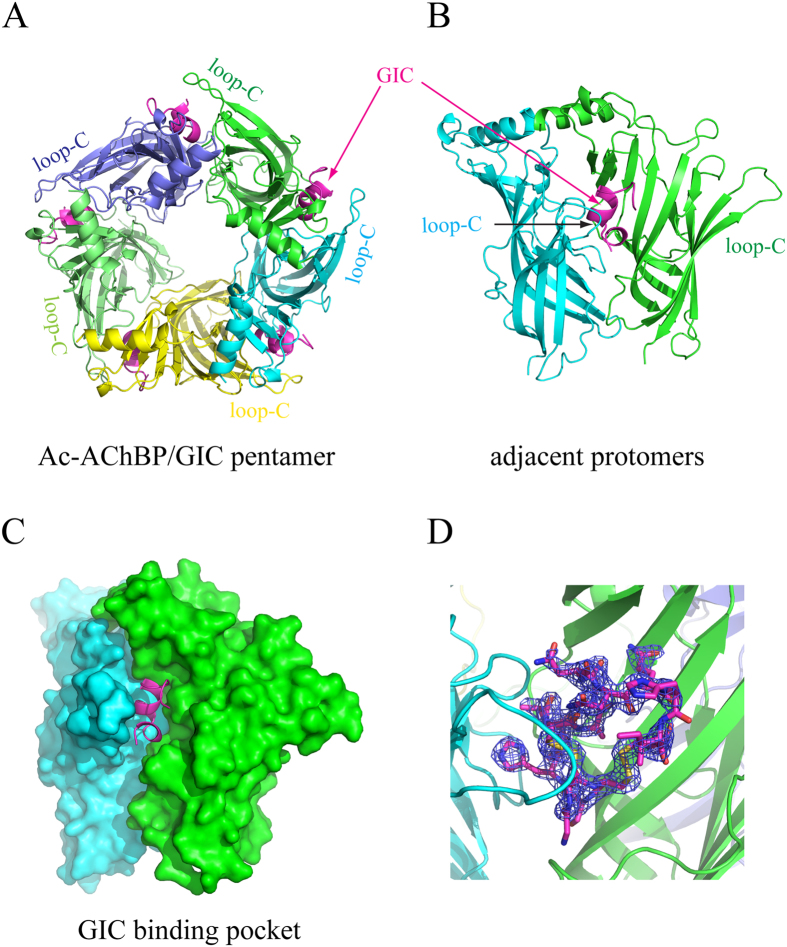
The X-ray crystal structure of *Ac*-AChBP in complex with α-conotoxin GIC. (**A)** The top view of the pentameric structure with five *Ac*-AChBP protomers, each in different colours and five α-conotoxin GIC molecules in magenta. (**B)** The side view of two adjacent protomers of the pentamer with a bound α-conotoxin GIC molecule (in magenta). (**C)** The side view of the surface model of two adjacent protomers with a bound α-conotoxin GIC molecule (in magenta) inside the binding pocket. (**D**) Fo-Fc electron density omit map contoured at 3.0 σ surrounding the GIC.

In the complex, the *Ac*-AChBP exists as a windmill-like pentamer bound with five α-conotoxin GIC peptides (Fig. 1A). Each ligand binding site in the *Ac*-AChBP is located between two adjacent protomers (Fig. 1B), and the five binding sites are structurally very similar due to the five-fold symmetry of the *Ac*-AChBP pentamer (Fig. 1A). In the complex, the bound α-conotoxins share a common orientation, with the central helix protruding into the ligand binding site of *Ac*-AChBP and the N and C termini of the bound α-conotoxin at the bottom and top parts of the ligand binding site, respectively (Fig. 1C,D).

**Figure 2:**
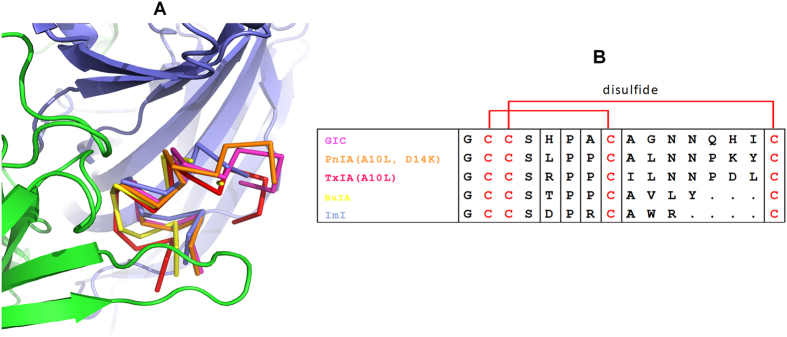
Comparison of different α-conotoxins bound by *Ac*-AChBP. (**A)** Backbone orientations observed in co-crystal structures of *Ac*-AChBP with five α-conotoxins. The backbone of GIC is shown in magenta, PnIA (A10L, D14K) in orange, ImI in light blue, TxIA (A10L) in red and BuIA in yellow. (**B)** Multiple sequence alignment of α-conotoxins GIC, PnIA (A10L, D14K), TxIA (A10L), ImI and BuIA. Disulfide bridges between Cys2-Cys8 and Cys3-Cys16 are shown in red.

The pentameric *Ac*-AChBP/GIC complex is similar to previously reported complexes with other α-conotoxins. Upon structural superimposition, the *Ac*-AChBP/GIC complex structure had a RMSD of 0.71 Å for all paired Cα atoms compared with the *Ac*-AChBP/PnIA (A10L, D14K) complex (PDB code 2BR8)^5^, 0.54 Å with the *Ac*-AChBP/ImI complex (PDB code 2C9T and 2BYP)^11^,^12^, 0.58 Å with the *Ac*-AChBP/TxIA (A10L) complex (PDB code 2UZ6)^13^ and 0.60 Å with the *Ac*-AChBP/BuIA complex (PDB code 4EZ1). All five different α-conotoxins displayed a similar binding mode with the central helix of the peptide protruding into the binding site of *Ac*-AChBP (Fig. 2A), but the detailed interactions are different due to amino acid differences among the peptides (Fig. 2B). The structure of GIC alone has been previously determined by an NMR method (PDB code 1UL2)^17^. The backbone of the GIC (residues 2–16) was structurally conserved in the unbound and *Ac*-AChBP-bound states with a RMSD of 0.53 Å in this region.

### Binding interface

**Figure 3:**
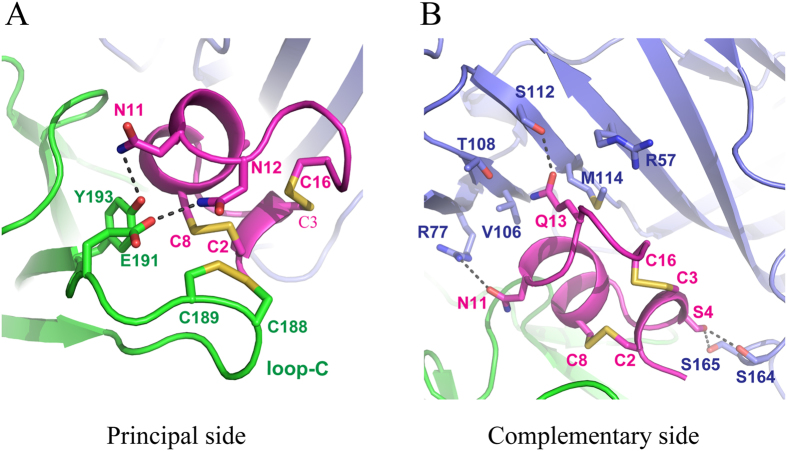
Binding interface between GIC and *Ac*-AChBP. **(A)** The disulfide bridge packing and hydrogen-bonding interactions (represented by black dashed line) on the principal side. Residues Asn-11 and Asn-12 of the GIC form hydrogen bonds with Tyr-193 and Glu-191 of the *Ac-*AChBP, respectively. Disulfide bond C2-C8 in the GIC closely packed together with C-188-C189 in the *Ac-*AChBP. (**B)** On the complementary side, the Gln-13 residue of GIC was located in a pocket surrounded by residues Arg-57, Val-106, Thr-108, Ser-112 and Met-114 of *Ac-*AChBP. Asn-11 of the GIC forms a hydrogen bond with Arg-77 of the *Ac-*AChBP. Ser-4 of the GIC also makes two hydrogen bonds with Ser-164 and Ser-165 of the *Ac-*AChBP.

The GIC interacts with two adjacent *Ac*-AChBP protomers at the interface, forming the principal and complementary binding sides (Fig. 1B). Most interactions on the principal side were between the GIC and C loop (Gln-184 ~ Tyr-193) of one of the *Ac*-AChBP protomers (Fig. 1B and Table 1). The chemical nature of interactions at this side is a mixture of hydrophobic and hydrophilic interactions. The Cys-2/Cys-8 disulfide bridge of the GIC stacked onto the vicinal Cys-188/Cys-189 disulfide bond of the *Ac*-AChBP (Fig. 3A and Table 1). Other significant hydrophobic interactions included the contacts of Trp-145 and Tyr-193 of the *Ac*-AChBP with surrounding GIC residues including His-5, Pro-6, Ala-7, Cys-8, Asn-11 and Asn-12 (Table 1). Hydrogen-bonding interactions occurred between Asn-11 and Asn-12 of the GIC with Tyr-193 and Glu-191 of the *Ac*-AChBP, respectively (Fig. 3A). The complementary binding side was between the GIC and β-sheet of another *Ac*-AChBP protomer in the interface (Table 1). On this side, a notable interacting residue in the GIC was Gln-13, whose side chain resided in a pocket consisting of residues contacts with Arg-57, Val-106, Thr-108, Ser-112 and Met-114 of *Ac*-AChBP (Fig. 3B). Gln-13 also formed a hydrogen bond with Ser-112 of *Ac*-AChBP (Fig. 3B). On this side, additional hydrogen-bonding interactions included GIC Ser-4 to *Ac*-AChBP Ser-164 and Ser-165 and GIC Asn-11 to *Ac*-AChBP Arg-77 (Fig. 3B).

### Molecular docking and modelling of GIC complexes with the α3β2 nAChR subtype

GIC potently blocks the α3β2 subtype of human nAChR, showing the highest known selectivity (100,000-fold selectivity for the α3β2 subtype vs the muscle receptor of nAChR) for neuronal versus muscle subtypes of any nicotinic ligand characterized to date^18^. Using the co-crystal structure of *Ac*-AChBP/GIC as a template, we first modelled the structures of the α3 and β2 subunits and then generated a α3β2/GIC complex model. The interacting residues of the GIC and the α3β2 nAChR are listed in Table 1. The GIC-contacting residues in the α3β2 nAChR and *Ac*-AChBP are very similar in both position and chemical property of the side chain (Table 1). One notable change is the substitution of Ser-148 in the *Ac*-AChBP with Asp-152 in the α3β2 nAChR. In the α3β2/GIC model, the α3 subunit Asp-152 has more extensive interactions with GIC Asn-11 than the *Ac*-AChBP Ser-148 does in the *Ac*-AChBP/GIC crystal structure. Another notable change is the substitution of Arg-57 in the *Ac*-AChBP with Glu-61 in the α3β2 nAChR. The residue change would not significantly affect the GIC binding because Arg-57 in the *Ac*-AChBP and Glu-61 in the α3β2 nAChR are just involved in the formation of the pocket for GIC Gln-13, and both residues have no specific interactions with GIC in the *Ac*-AChBP/GIC crystal structure and α3β2/GIC model.

**Figure 4:**
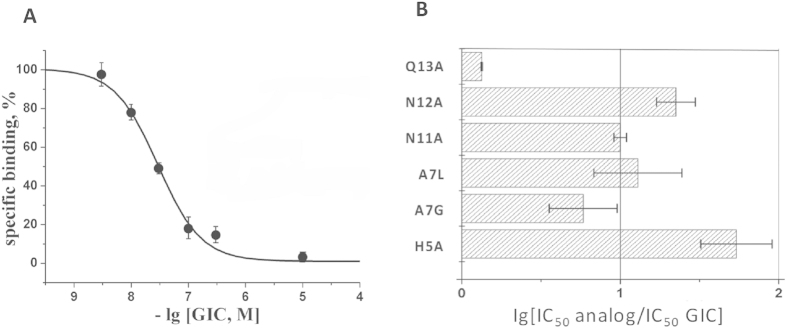
The binding affinity of α-conotoxin GIC and its mutants to *Ac*-AChBP measured by a competitive radioligand assay. (**A)** The inhibition curve for α-conotoxin GIC wild-type is shown. Each point is a mean ± s.e.m value of two measurements for each concentration of one experiment. The curve was calculated from the means ± s.e.m. using the ORIGIN 7.5 program. The calculated IC_50_ value is 29 ± 2 nM. (**B)** The bar diagram showing the differences in IC_50_ values for GIC mutants in comparison with wild-type peptides.

According to the structural and docking data, GIC His-5, Ala-7, Asn-11, Asn-12 and Gln-13 are residues that form the largest number of interactions with the *Ac*-AChBP or α3β2 nAChR (Table 1). Therefore, we chose these five sites and synthesized six GIC analogues (i.e., His5Ala, Ala7Gly, Ala7Leu, Asn11Ala, Asn12Ala and Gln13Ala) with single-site mutations in each analogue (Table 2). The wild-type GIC and these six analogues were synthesized by solid-phase peptide synthesis. The characterization of the synthetic GIC mutants by reverse-phase high performance liquid chromatography (HPLC) and mass spectrometry are shown in Supplementary Figure 1. All α-conotoxin GIC and its mutant peptides have CD spectra consistent with the CysI-III, CysII-IV disulphide isomer containing a helical character (See Supplementary Figure 2). We first studied the binding of these GIC analogues (in comparison with wild-type GIC) towards the *Ac*-AChBP by measuring their abilities to compete with [^125^I]-labelled α-bungarotoxin in a radioligand assay. In the dose response experiment, the wild-type GIC exhibited a complete inhibition of radioligand binding with IC_50_ = 29 ± 2 nM (Fig. 4A). Among the studied GIC mutants, only the Gln13Ala substitution essentially did not change the affinity for *A*c-AChBP (Fig. 4B). The substitutions of Ala-7, Asn-11 and Asn-12 in GIC resulted in approximately one order drop in the binding affinity for *Ac*-AChBP (Fig. 4B). The most significant change was observed for the His5Ala mutation with an almost 100-fold increase in the IC_50_ value (Fig. 4B). We then tested the inhibition of the α3β2 nAChR by these GIC mutants in an electrophysiology experiment. All six GIC analogues except Gln13Ala showed a very significant decrease in the inhibition of the α3β2 nAChR compared with wild-type GIC (Table 2). The GIC analogues bearing the Ala7Leu or His5Ala mutation exhibited a more than 10,000-fold decrease in the inhibition (Table 2). These data further support our α3β2/GIC model built on the *Ac*-AChBP/GIC crystal structure.

### Potential structural basis for the high selectivity of GIC towards the α3β2 subtype

**Figure 5:**
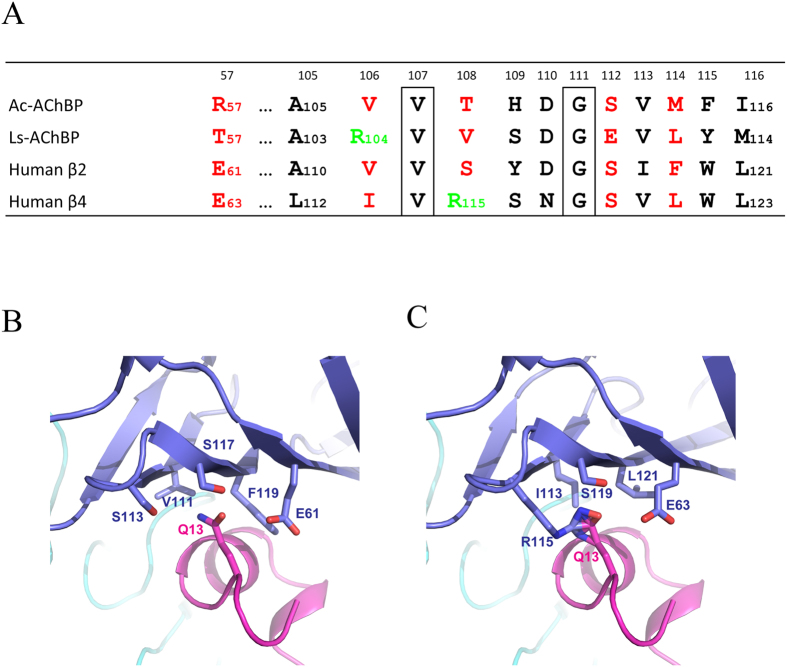
Structure basis for the selectivity of GIC with α3β2 vs α3β4 nAChR. (**A)** Primary sequence comparison of *Ac*-AChBP, *Ls*-AChBP, human β2 and human β4 residues forming the pocket on the complementary binding side to accommodate Gln-13 of the GIC. (**B)** The pocket in human β2 is able to accommodate Gln-13 of the GIC without steric clashes in the α3β2/GIC model. (**C)** Arg-115 in the pocket of human β4 could bring steric hindrance and even steric clashes with Gln-13 of the GIC and then disfavour the binding of α3β4 compared with α3β2 nAChR.

GIC exhibits a high affinity (~1 nM) to the α3β2 nAChR, but a much lower (~700 nM) affinity towards the α3β4 nAChR (Table 2). Sequence alignment of *Ac*-AChBP, AChBP from *Lymnaea stagnalis* (*Ls*-AChBP), as well as of human β2 and β4 nAChR subunits, are shown in Supplementary Fig. 3. The regions of nAChR subunits forming the binding sites are shown in Fig. 5A (summary from Supplementary Figure 3). With the structure of *Ac*-AChBP/GIC as a template, we also docked the GIC onto human α3β4 nAChR models. The comparison of the α3β2/GIC model, α3β4/GIC model and *Ac*-AChBP/GIC crystal structure allowed us to pinpoint the key amino acid residues on the complementary side, which appear to be responsible for selectivity of GIC for α3β2 vs α3β4. In the GIC/α3β2 model, the Gln-13 of GIC resides in a pocket surrounded by Glu-61, Val-111, Ser-113, Ser-117 and Phe-119 residues of the β2 subunit (Fig. 5B), which is very similar to the respective pocket formed by Arg-57, Val-106, Thr-108, Ser-112 and Met-114 residues on the complementary side of *Ac*-AChBP (Fig. 3B). No steric clashes were found between Gln-13 of GIC and the α3β2 receptor in the model, thus providing an explanation for a high affinity to both *Ac*-AChBP and the α3β2 receptor. Residues forming the pocket in the β4 subunit were changed to Glu-62, Ile-113, Arg-115, Ser-119 and Leu-121, which, except for Arg-115, were the same or similar to the respective residues in the β2 subunit (Fig. 5A). This position in the *Ac*-AChBP and *Ls*-AChBP are threonine and valine, respectively (Fig. 5A). Arg-115, which has a longer side chain compared to serine, threonine and valine, could bring steric hindrance in this pocket to accommodate Gln-13 of GIC and could even directly clash with it (Fig. 5C). The presence of arginine at this position would not favour the binding of the GIC with the α3β4 nAChR compared with the α3β2 nAChR, which is also represented by the decreased inhibition ability of GIC against the α3β4 receptor (Table 2).

## Discussion

Before this study, co-crystal structures of four different α-conotoxins in complexes with *Ac*-AChBP have been reported, namely for the PnIA variant (PDB code 2BR8)^5^, TxIA variant (PDB code 2UZ6)^13^, ImI (PDB code 2C9T and 2BYP)^12^ and BuIA (PDB code 4EZ1). All *Ac*-AChBP/α-conotoxin co-crystal structures are similar, but the residues interacting at the binding sites are different (Supplementary Table 2). As a result, different α-conotoxins exhibit a wide range in selectivity towards distinct nAChR subtypes, reflected in different pharmacological properties (Supplementary Table 3). The *Ac*-AChBP/TxIA (A10L) co-crystal structure showed that the Arg-5 of TxIA (A10L) forms a hydrogen bond with Tyr-186 and a salt bridge with Asp-195 residues of loop C, which account for the high binding affinity of this conotoxin variant to *Ac*-AChBP. A docking study of TxIA (A10L) onto α3β2 nAChR suggested that Arg-5 could have a salt bridge with Asp-197 and a hydrogen bond with Tyr-188 of the α3 subunit (corresponding to Asp-195 and Tyr-186 in Ac-AChBP)^13^. The *Ac*-AChBP/ImI co-crystal structure indicated that α-conotoxin Trp-10 residue could play a key role in binding to α3β2 nAChR. Substitution of Thr-57 in the β2 subunit by Lys-59 in the β4 subunit would result in steric clashes with the Trp-10 residue of ImI, which explains the decreased affinity for the α3β4 subtype^7^,^12^. α-Conotoxin GIC is one more α-conotoxin of strong selectivity for α3β2 nAChR. Compared with TxIA(A10L) and ImI, GIC does not contain the same respective Arg-5 or Trp-10 residues responsible for α3β2 binding and selectivity. Instead, it has His-5 and Gln-13 residues in these two positions.

Through the co-crystal structure of *Ac*-AChBP/GIC and the GIC docking to different nAChR subtypes, we came to the conclusion that His-5 and Gln-13 of GIC are important residues for its α3β2 nAChR binding and selectivity, respectively. These conclusions were further supported by the studies of GIC analogues in *Ac*-AChBP binding and α3β2 nAChR inhibition. The radioligand binding and electrophysiology experiments were different in sensitivity, but the results from both experiments revealed a common tendency: the His5Ala mutant showed the largest drop in ability for *Ac*-AChBP binding and for the α3β2 nAChR functional blocking, and the Gln13Ala substitution had the least influence on GIC activity in both experiments (Fig. 4B and Table 2). In accordance with the co-crystal structure, the His5Ala mutation led to a dramatic (by almost 2 orders of magnitude) decrease in affinity for *Ac*-AChBP. As shown in Table 1, the His-5 interacted extensively with Tyr-91, Tyr-186 and Tyr-193 of the *Ac*-AChBP and its mutation to Ala would lead to less interaction and decreased binding. In the α3β2/GIC model, His-5 of GIC also interacted with three tyrosines (i.e., Tyr-93, Tyr-190 and Tyr-197) of the α3 subunit. Therefore, its mutation to alanine would decrease its inhibitory effect by reducing the interactions with surrounding tyrosine residues in the α3 subunit. Similar to the His-5 position, substitutions of amino acid residues in Ala-7, Asn-11 and Asn-12 positions involved in the interactions on the principal side also had a significant impact on the activity of GIC in both assays (Fig. 4B and Table 2). Asn-11 and Asn-12 participate in the interaction with *Ac*-AChBP by forming hydrogen bonds with Glu-191 and Tyr-193 (Fig. 3A), and these two residues in *Ac*-AChBP were conserved in the α3 subunit. Ala-7 in GIC was different from the proline in PnIA, TxIA and BuIA and arginine in ImI. It formed hydrophobic interactions with Trp-145, Val-146 and Tyr-193 of *Ac*-AChBP. A change to leucine or glycine seems to break these interactions and thus reduce the activity of GIC (Fig. 4B and Table 2). These results, together with previous studies on other α3β2 nAChR selective α-conotoxins, suggested that the high affinity and specificity of α-conotoxin to the α3β2 nAChR subtype is determined basically by its interactions with the principal side of this receptor^20^.

In contrast, for Gln-13, which resides in a pocket formed by residues Arg-57, Val-106, Thr-108, Ser-112 and Met-114 on the complementary side of *Ac*-AChBP (Fig. 3B), its replacement for alanine did not significantly affect the activity in both assays (Fig. 4B and Table 2). Because the binding of GIC with *Ac*-AChBP or α3β2 nAChR is basically determined by multiple interactions on the principal side, a mutation of Gln-13, which is involved in the interactions on the complementary side, would not significantly affect the activity of GIC in *Ac*-AChBP binding and α3β2 nAChR inhibition. Previous studies have proposed that α-conotoxin residues on the complementary side, such as the Trp-10 in ImI, are important for the receptor selectivity of the toxin. Our docking studies with α3β2 vs α3β4 models also supported this suggestion. In the α3β2/GIC model, no steric clashes were found between Gln-13 of GIC and the β2 receptor subunit, which had a similar pocket consisting of Glu-61, Val-111, Ser-113, Ser-117 and Phe-119 (Fig. 5B). It provided an explanation for high affinity to both *Ac*-AChBP and α3β2 receptors by GIC. In contrast, the Arg-115 residue of the human β4 subunit, instead of threonine in *Ac*-AChBP and serine in the human β2 subunit (Fig. 5A), could have steric clashes with Gln-13 residues of GIC (Fig. 5C), which may explain the low affinity of GIC towards the α3β4 receptor subtype and its high selectivity of α3β2 vs α3β4. Our data from the co-crystal structure may also explain why *Ls*-AChBP shows a 10-fold decreased affinity for GIC in comparison with *Ac*-AChBP (Supplementary Table 3): the Arg-104 residue of *Ls*-AChBP (Val-106 *Ac*-AChBP) could also have steric clashes with the Gln-13 residue of GIC on the complementary side (Fig. 5A).

The models of α-conotoxin GIC complexes with α3β2 and α3β4 nAChRs were previously generated using methods of molecular docking 19. These models suggested that the GIC’s higher affinity for α3β2 vs α3β4 was due to the residues in the α3β2 subtype that are more closely located to the ligand than the respective residues in the α3β4 subtype. However, why the residues in the α3β2 subtype are situated more closely to GIC has not been explained. Our model based on the AChBP co-crystal structure allowed us to conclude that the major reason for α3β4 decreased affinity for GIC is probably the steric clashes between the Arg-115 residue of the β4 subunit and the peptide Gln-13 residue.

Notably, none of the substitutions led to an increase in the GIC affinity for α3β4 nAChR (Table 2). In general, our results demonstrated that more sophisticated computer methods are required to correctly transfer the data from the co-crystal structure of α-conotoxin or any other ligand, with a model like *Ac*-AChBP, to a true receptor. Even for the model as such, from the two most important GIC residues (His-5 and Gln-13), in our view, we experimentally could confirm only the role of His-5. This residue was also shown to be essential for recognition of α3β2 nAChR. It also should be noted that GIC has a high affinity both for *Ac*-AChBP and α3β2 nAChR, and the importance of one or another GIC residue was demonstrated by a decrease in the affinity of the chosen mutants. However, in the case of α3β4 nAChR, the task was the opposite: it was expected that the chosen GIC substitutions will raise the affinity to those subtypes. In general, increasing the affinity (or activity) is always a much more challenging task. Apparently, the co-crystal structure of GIC with *Ac*-AChBP is a good starting point and only extensive subsequent creation of substituted α-conotoxin libraries and a broad mutagenesis of the particular nAChR of interest will unravel the fine mechanisms of binding and might assist the design of more potent and selective antagonists and agonists.

In summary, we first determined the crystal structure of GIC with *Ac*-AChBP. Based on this structure, we obtained a more accurate model of α-conotoxin GIC complexed with α3β2 and also presented possible explanations for the high selectivity of GIC for α3β2. We believe that these results will help develop a better understanding of the α-conotoxin selectivity for distinct nAChR subtypes and may facilitate the design of α-conotoxin analogues for therapeutic purposes.

## Materials and Methods

### Chemical synthesis of GIC and its mutants

The linear peptides were assembled by solid-phase methodology on an ABI 433A peptide synthesizer (Applied Biosystems Inc., Foster City, CA, USA) using FastMoc [N-(9-fluorenyl)methoxycarbonyl] chemistry and standard side-chain protection, except for cysteine residues. A two-step oxidation protocol was used to fold the peptide selectively, as described previously^20^. Briefly, to form first disulfide bridge between Cys2 and Cys8, the peptides were added slowly to an equal volume of 20 mM potassium ferricyanide K_3_[Fe(CN)_6_] and 0.1 M Tris base, with the pH adjusted to 7.5 with acetic acid. The solution was mixed to react for 45 min, and the monocyclic peptide was purified by reverse-phase HPLC. Simultaneous removal of the S-acetamidomethyl groups and closure of the disulfide bridge between Cys3 and Cys16 was carried out by iodine oxidation, the bicyclic peptide was purified by HPLC on a reversed-phase C18 Vydac column (Hesperia, CA, USA) using a linear gradient of 10–40% B90 in 30 min. Solvent B was 90% ACN, 0.092% TFA, and H_2_O; Solvent A was 0.1% TFA in H_2_O. Analytical reversed phase-HPLC (Waters ACQUITY UPLC H-Class) and ESI -IT-TOF (Shimadzu, Kyoto, Japan) mass spectrometry confirmed the purity and molecular mass of the synthesized peptides. The purity and correctness of the structure of synthesized products were checked by HPLC re-chromatography and ESI mass-spectrometry (Fig. 1 Supplementary).

### Protein expression and purification

The *Ac*-AChBP was expressed using the Bac-to-Bac baculovirus expression system (Invitrogen). Spodoptera frugiperda Sf9 insect cells were maintained in Insect-Xpress protein-free medium (Lonza) without serum. Full length *Ac*-AChBP with a C-terminal 6× His tag was cloned into the pFastBac-Dual vector (Invitrogen). The construct was transformed into bacterial DH10Bac competent cells, and the extracted bacmid was transfected into Sf9 cells using Cellfectin II Reagent (Invitrogen). The low-titre viruses (P0) were harvested after incubation of the transfected cells at 26 °C for 7 ~ 9 d and then amplified to generate a high-titre virus stock. The amplified high-titre viruses (P1) were used to infect 1 L of Sf9 cells at a density of 2 × 10^6^ cells/ml. The supernatant of the cell culture containing soluble *Ac*-AChBP was harvested 48 h after infection and concentrated and buffer-exchanged to HBS (10 mM Hepes, pH 7.2, 150 mM NaCl). *Ac*-AChBP was captured by Nickel-charged resin (GE Healthcare) and eluted with 500 mM imidazole in HBS buffer (pH 7.2). Further purification was performed by gel-filtration chromatography using the Superdex 200 column (GE Healthcare).

### Crystallization and data collection

Purified *Ac*-AChBP and synthesized α-conotoxin GIC were mixed at a molar ratio 1:1.5 at 4 °C for 2 h and then loaded to a Superdex 200 column. The complex was collected and concentrated to ~20 mg/ml in HBS buffer for crystallization. Crystals were successfully grown at 18 °C using the sitting drop vapour diffusion method by mixing equal volumes of protein and reservoir solution containing 1.5 M lithium sulfate monohydrate, 0.1 M Tris, PH 8.5. Crystals were cryocooled in liquid nitrogen with cryoprotectant (reservoir solution plus 20% (v/v) glycol) before data collection. Diffraction data were collected at the BL17U beam line of the Shanghai Synchrotron Research Facility (SSRF). Diffraction data were indexed, integrated and scaled with the program HKL2000^21^.

### Structural determination and refinement

The structure was determined by the molecular replacement method with PHASER^22^ in CCP4 suite^23^. The search model was Ac-AChBP–PnIA (D14K, A10L) (PDB code 2BR8). Iterative structural refinement was performed with the program PHENIX^24^ and the structure validation was performed by the program PROCHECK^25^. All structural figures were made with PyMol^26^.

### Molecular modelling, docking and simulations

All the modelling, docking and simulations were performed in Discovery Studio Client 4.0 (Accelrys, San Diego, CA). The molecular models of extracellular ligand-binding domains of the human nAChRs such as (α3)_2_(β4)_3_, (α3)_2_(β2)_3_ were generated based on the template of *Ac*-AChBP structure using the homology modelling program Modeler version 9.0. The GIC docking was based on the reference model of the *Ac*-AChBP/GIC complex. The models were refined with a side-chain refinement and energy minimization process. The CHARMm forcefield was used for all simulations. All modelling and docking structures were verified by the program Profiles-3D in the Discovery Studio platform^27^, as well as by the MolProbity server^28^,^29^.

### Competitive radioligand assay

For competitive radioligand assays we used 6× His tagged *Ac*-AChBP and *Ls*-AChBP, kindly provided by Prof. A.B. Smit (Free University of Amsterdam, the Netherlands), which were expressed and purified mainly as described above for *Ac*-AChBP. The competition experiments with [^125^I]-labelled α-bungarotoxin were carried out mainly as described in ref. 30. Shortly, α-conotoxin GIC or its mutants (in a concentration range of 0.03–100 μM) were pre-incubated 3 h at room temperature with the *Ls-* or *Ac-*AChBPs (the final protein concentration of 2.4 nM or 140 nM, respectively) in 50 μL of binding buffer (20 mM Tris-HCl buffer, 1 mg/mL of bovine serum albumin, pH 7.9). Next, [^125^I]- labelled α-bungarotoxin was added to final concentration 0.2 nM and the mixtures were additionally incubated for 5 min. Binding was stopped by rapid filtration on double DE-81 filters (Whatman) pre-soaked in binding buffer, unbound radioactivity being removed from the filters by washout (3 × 3 mL) with the binding buffer. Non-specific binding was determined in all cases using 3 h pre-incubation with 10 μM α-cobratoxin from *Naja kaouthia* venom.

The binding results were analysed using ORIGIN 7.5 (OriginLab Corporation, Northampton, MA, USA) fitting to a one-site dose-response curve by the equation: % response = 100/{1 + ([toxin]/IC_50_)^*n*^, where IC_50_ is the concentration at which 50% of the binding sites are inhibited and *n* is the Hill coefficient^30^.

### Electrophysiology measurements and data analysis

The cRNAs of nAChR subunits (kindly provided by S. Heinemann, Salk Institute, San Diego, CA, USA) were obtained by *in vitro* transcription using the mMessage mMachine SP6 kit (Ambion, Austin, TX, USA). The MEGAclearTM kit (Ambion) was used to purify the cRNAs. Oocytes of *Xenopus laevis* were prepared and injected with capped RNA (cRNA) to express human α3β2, and human α3β4 nAChRs. Oocytes were injected within one day of harvesting and recordings were made 1–4 days post-injection, as described previously^18^. Briefly, oocytes were transferred to the recording chamber (~50 μL in volume) and gravity-perfused at 2 mL/min with ND-96 buffer containing 0.1 mg/mL bovine serum albumin (BSA). ACh-gated currents were obtained with a two-electrode voltage-clamp amplifier (Axoclamp 900A, Molecular Devices Corp., Sunnyvale, CA, USA). Oocytes were voltage clamped at −70 mV at room temperature. The continuous gravity perfused with standard ND-96 solution and stimulated with 2-s pulses of ACh once every minute. A total of 5 μL of different concentration toxins were placed in the chamber for 5 min, and then the perfusion system was applied during which 2-s pulses of 100 μM ACh were applied every minute until a constant level of blockage was achieved. The final results were acquired for at least 4 oocytes. The dose-response data were fitted to the equation: % Response = 100/{1 + ([toxin]/IC_50_)n_H_}, Each data point of the dose-response curve represents the average ± S.E. of 4 to 6 oocytes, where n_H_ is the Hill coefficient, by nonlinear regression analysis using GraphPad Prism (GraphPad Software, San Diego, CA, USA)^18^.

### PDB deposition

The coordinates and diffraction data have been deposited into the Protein Data Bank with accession code 5CO5.

## Additional Information

**How to cite this article**: Lin, B. *et al.* From crystal structure of α-conotoxin GIC in complex with *Ac*-AChBP to molecular determinants of its high selectivity for α3β2 nAChR. *Sci. Rep.* **6**, 22349; doi: 10.1038/srep22349 (2016).

## Supplementary Material

Supplementary Information

## Figures and Tables

**Table 1:** Amino acid contacts with a distance cutoff 4.0 Å between α-conotoxin GIC and *Ac*-AChBP from the X-ray structure or α3β2 nAChR from modelling.

GIC/Ac-AChBP crystal structure		GIC/α3β2-nAChR model	
Principal side			
GIC	*Ac*-AChBP	GIC	α3β2
His-5	Tyr-91	His-5	Tyr-93
Pro-6, Ala-7	Trp-145	Pro-6, Ala-7	Trp-149
Ala-7	Val-146	Ala-7	Ser-150
Asn-11	Ser-148	Ala-7, Asn-11	Asp-152
Gly-1, Cys-2, His-5, Cys-8	Tyr-186	Gly-1, Cys-2, His-5, Cys-8	Tyr-190
Cys-2	Cys-188	Asn-12, Ile-15	Cys-192
Asn-12	Cys-189	Asn-12	Cys-193
Asn-11, Asn-12	Glu-191	Asn-11, Asn-12	Glu-195
His-5, Ala-7, Cys-8, Asn-11, Asn-12	Tyr-193	His-5, Cys-8, Asn12	Tyr-197
Complementary side			
Ser-4	Thr-34	Ser-4	Ser-38
Ser-4, Pro-6	Tyr-53	Ser-4, His-5, Pro-6	Trp-57
Ala-9, Cys-16	Gln-55		
Gln-13	Arg-57	Gln-13, Cys-16	Glu-61
Asn-11	Arg-77	Asn-11	Arg-81
Gly-10, Gln-13	Val-106	Gly-10, Gln-13	Val-111
Gln-13	Thr-108		
Gln-13	Ser-112	Gln-13	Ser-117
Ala-9, Gly-10, Gln-13	Met-114	Ala-9, Gly-10, Gln-13	Phe-119
Pro-6, Ala-9	IIe-116		Leu-121
Cys-16	Asp-157		
Ser-4	Asp-162, Ser-164, Ser-165	Ser-4	Asp-171

**Table 2:** Amino acid sequences and blocking activities (in IC_50_s, nM) of α-conotoxin GIC and its analogues on expressed nAChR subtypes.

Peptide	Sequence	Subtype	IC_50_ (nM)	Hill Slope	Subtype	IC_50_ (nM)
GIC	GCCSHPACAGNNQHIC*	hα3β2	1.13(1.10–1.16)	0.764(0.559–0.969)	hα3β4	750(710–790)
GIC(Q13A)	GCCSHPACAGNNAHIC*	hα3β2	8.41(7.027–10.01)	1.078(0.823–1.332)	hα3β4	660(630–690)
GIC(N12A)	GCCSHPACAGNAQHIC*	hα3β2	1980(840–4660)	0.35(0.238–0.463)	hα3β4	>10000
GIC(N11A)	GCCSHPACAGANQHIC*	hα3β2	1600(545–4720)	0.491(0.222–0.760)	hα3β4	>10000
GIC(A7L)	GCCSHPLCAGNNQHIC*	hα3β2	>10000	—	hα3β4	>10000
GIC(A7G)	GCCSHPGCAGNNQHIC*	hα3β2	4210(1566–11330)	0.371(0.222–0.521)	hα3β4	>10000
GIC(H5A)	GCCSAPACAGNNQHIC*	hα3β2	>10000	—	hα3β4	>10000

^*^C-terminal carboxamide.
