# Supplementary material for: From crystal structure of α-conotoxin GIC in complex with Ac-AChBP to molecular determinants of its high selectivity for α3β2 nAChR
Source: Sci Rep. 2016 Mar 1;6:22349. doi: 10.1038/srep22349 (PMC4772116; doi:10.1038/srep22349)
Supplement: Supplementary Information [file srep22349-s1.pdf]

Supplementary information

From crystal structure of α-conotoxin GIC in complex with *Ac*-AChBP to molecular determinants of its high selectivity for α3β2 nAChR

Bo Lin[1,#], Manyu Xu[2,#], Xiaopeng Zhu[1], Yong Wu[1], Xi Liu[2], Dongting Zhangsun[1], Yuanyan Hu[1], Shi-Hua Xiang[4], Igor E. Kasheverov[3], Victor I. Tsetlin[3], Xinquan Wang[2,]* and Sulan Luo[1,]*

[1]Key Laboratory of Tropical Biological Resources, Ministry of Education, Key Lab for Marine Drugs of Haikou, Hainan University, Haikou Hainan 570228, P. R. China.
[2]Ministry of Education Key Laboratory of Protein Science, Center for Structural Biology, School of Life Sciences, Tsinghua University, Beijing 100084, P. R. China.
[3]Shemyakin-Ovchinnikov Institute of Bioorganic Chemistry, Russian Academy of Sciences, Miklukho-Maklaya Street, 16/10 Moscow 117997, Russia.
[4]Nebraska Center for Virology, School of Veterinary Medicine and Biological Sciences, University of Nebraska-Lincoln, Lincoln, NE 68583, USA.

[#]Equal contribution

***Corresponding authors:**
Sulan Luo, Ph.D.
Key Laboratory of Tropical Biological Resources, Ministry of Education, Hainan University, Haikou Hainan 570228, P. R. China
Phone: (86) 898 66289538; Fax: (86) 898 66276720.
E-mail: luosulan2003@163.com

Xinquan Wang, Ph.D.
Ministry of Education Key Laboratory of Protein Science, Center for Structural Biology, School of Life Sciences, Tsinghua University, Beijing 100084, P. R. China.
E-mail: xinquanwang@mail.tsinghua.edu.cn

Running Title: Co-crystal structure of α-conotoxin GIC and acetylcholine binding proteins

Table S1|Crystal diffraction data collection and structural refinement statistics

**Data collection**	
Beamline	SSRF BL17U
Wavelength	0.9796 Å
Space group	$P2_12_12_1$
**Cell dimensions**	
a, b, c (Å)	78.6, 84.9, 208.6
**α, β, γ (°)**	90.0, 90.0, 90.0
Resolution (Å)	50-2.1
R_{merge} (%)	8.8 (67.7)
$I / \sigma I$	18.7 (3.5)
Completeness (%)	96.9 (93.5)
Redundancy	6.7 (6.9)
**Refinement**	
Resolution (Å)	43.5-2.1
No. Reflections	85409
R_{work} / R_{free} (%)	19.7/23.7
**No. atoms**	
Protein	8299
Ligand/ion	545
Water	521
**B factor (Å^2)**	
Protein	41.1
Ligand/ion	45.3
Water	42.5
**r.m.s. deviations**	
Bond lengths (Å)	0.008
Bond angles (°)	1.104

Table S2 | Contacts between *Ac*-AChBP and α-conotoxins (d < 4 Å)

Ac-AChBP	GIC [a]	PnIA(A10L, D14K) [b]	TxIA(A10L) [c]	ImI [d]	BulA
Principal side					
**Lys-23**				Arg-11	
**Tyr-91**	His-5	Leu-5, Pro-7	Arg-5, Pro-6, Pro-7	Arg-7	Pro-7
**Ser-144**		Pro-7	Pro-7	Arg-7	Pro-7
**Trp-145**	Pro-6, Ala-7	Pro-6, Pro-7	Pro-6, Pro-7	Pro-6, Arg-7	Pro-6, Pro-7
**Val-146**	Ala-7	Leu-10, Asn-11	Pro-7, Leu-10	Arg-7	Pro-7
**Tyr-147**			Pro-7	Arg-7	Pro-7
**Ser148**	Asn-11	Asn-11	Asn-11		
**Glu151**		Asn-11			
**Tyr-186**	Gly-1, Cys-2, His-5, Cys-8	Gly-1, Cys-2, Leu-5, Cys-8	Gly-1, Cys-2, Arg-5	Gly-1,Cys-2, Asp-5	Gly-1, Cys-2, Thr-5
**Cys-188**	Cys-2	Cys-2, Tyr-15	Cys-2	Cys-2	Cys-2
**Cys-189**	Asn-12	Cys-2, Asn-12	Cys-2, Asn-12	Cys-2	Cys-2, Tyr-12
**Glu-191**	Asn-11, Asn-12	Asn-12	Asn-11, Asn-12	Arg-11	Leu-11, Tyr-12
**Tyr-193**	His-5, Ala-7, Cys-8, Asn-11, Asn-12	Leu-5, Pro-7, Cys-8, Asn-11, Asn-12	Arg-5, Pro-7, Cys-8, Asn-11, Asn-12	Arg-7, Cys-8	Pro-7, Cys-8, Tyr-12
**Ile-194**				Arg-7	
**Asp-195**			Arg-5		
Complementary side					
**Thr-34**	Ser-4		Cys-3		Cys-3

**Tyr-53**	Ser-4, Pro-6	Pro-6	Ser-4, Pro-6	Ser-4	Ser-4
**Gln-55**	Ala-9, Cys-16		Ile-9	Cys-3	Cys-3, Ala-9, Cys-13
**Arg-57**	Gln-13,	Pro-13	Pro-13		Cys-13
**Asp-75**				Trp-10	
**Arg-77**	Asn-11	Asn-11	Asn-11	Trp-10	
**Ile-104**		Leu-10			
**Val-106**	Gly-10, Gln-13	Leu-10	Leu-10	Trp-10	Val-10
**Thr-108**	Gln-13			Trp-10	
**Asp-110**		Lys-14			
**Ser-112**	Gln-13	Lys-14			
**Met-114**	Ala-9,Gly-10, Gln-13	Ala-9, Leu-10, Pro-13	Ile-9, Leu-10, Pro-13	Trp-10	Val-10, Leu-11
**Ile-116**	Pro-6, Ala-9	Pro-6, Ala-9, Leu-10,	Leu-10	Pro-6, Ala-9	Ala-9, Val-10
**Asp-157**	Cys-16				
**Asp-162**	Ser-4	Ser-4	Cys-3	Ser-4	Cys-3
**Ser-164**	Ser-4	Ser-4	Ser-4	Gly-1, Ser-4	Ser-4
**Ser-165**	Ser-4		Ser-4		Ser-4

Table S3 | Affinity (as K_i or IC_{50}s, nM) of four α-conotoxins for *Ac*-AChBP, AChBP from *Lymnaea stagnalis* (*Ls*-AChBP) and different human or rat nAChR subtypes

α-conotoxins	*Ac*-AChBP	*Ls*-AchBP	hα3β2	hα3β4	hα7	rα3β2	rα7
GIC	29 ± 2 [a]	220 ± 10 [a]	1 [b]	755 [b]			
ImI	33 ± 5 [c]	4140 ± 400 [c]	41 [d]	3390 [d]	132 [e]	>5000 [f]	69 [g]
PnIA(A10L,D14K)	28 ± 6 [h]	13 ± 2 [h]			260 [h]		
TxIA(A10L)		1.1 [i]				2.0 [i]	39 [i]

Data from [a] this work, [b] ref. [11], [c] ref. [9], [d] ref. [21], [e] ref. [28], [f] ref. [22], [g] ref. [30], [h] ref. [7] and [i] ref.[10].

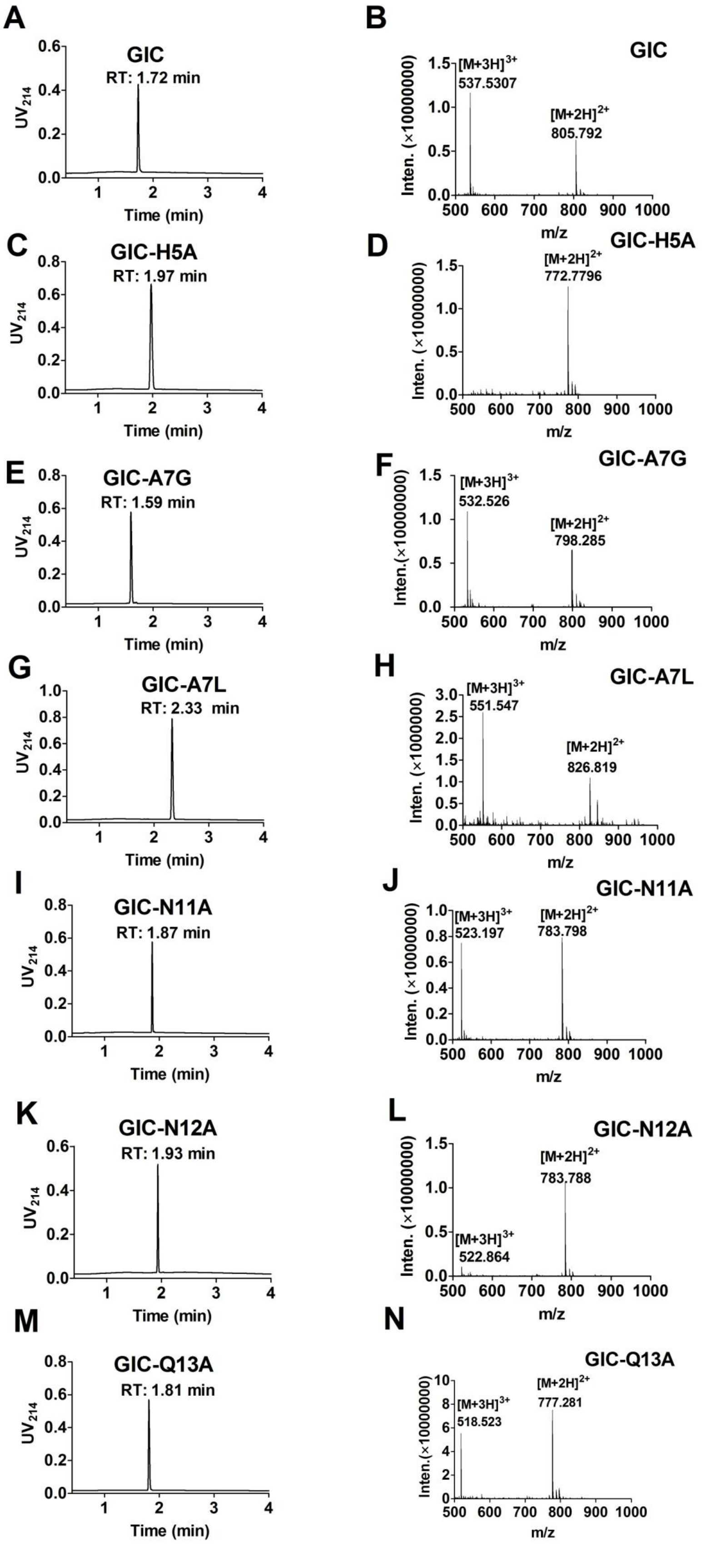

Figure S1| HPLC chromatograms with a retention time and ESI-MS data of

α-conotoxin GIC and its analogues. The peptides were analyzed on a waters ACQUITY UPLC BEH C18 column (2.1×50 mm, 1.7 μm) using a linear gradient of 5% Buffer B to 40% Buffer B over 3.5 min with flow rate of 0.5 ml/min at temperature of 40 ℃, where A = 0.1% trifluoroacetic acid (TFA) and B = 0.75% TFA, 90% acetonitrile, and the remainder water. Absorbance was monitored at 214 nm.

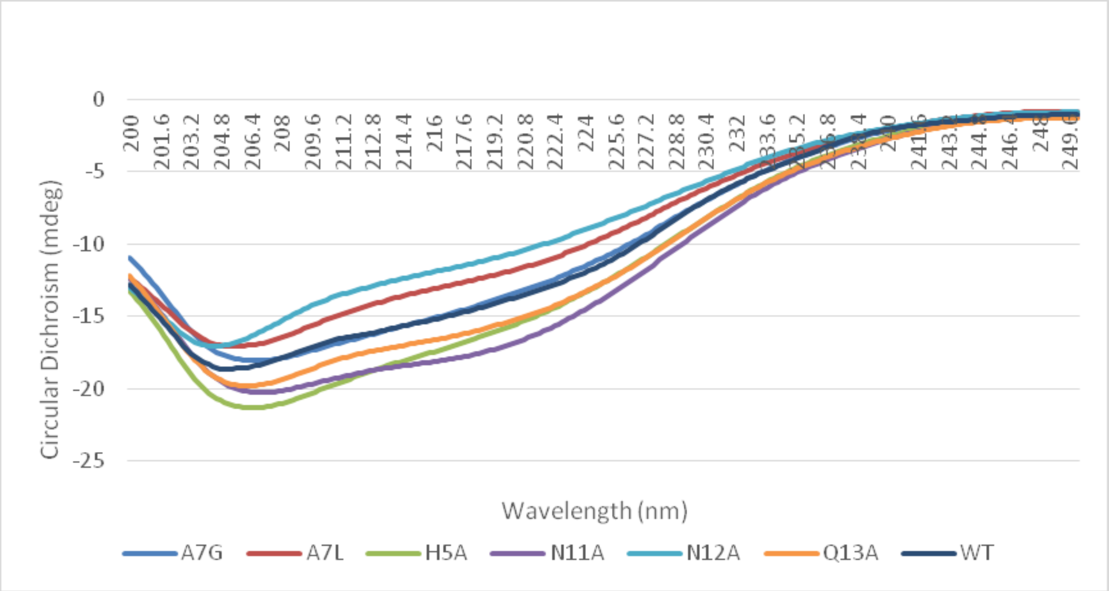

Figure S2| Circular dichroism of α-conotoxin GIC and its analogues. CD spectra were recorded on a JASCO J-715 Spectropolarimeter. Spectra was recorded at room temperature under nitrogen atmosphere. Peptides were dissolved to 100 μM. Spectra were recorded over a 200-250 nm range at 25 ℃ using an average of 3 scans (scan speed of 200 nm/min).

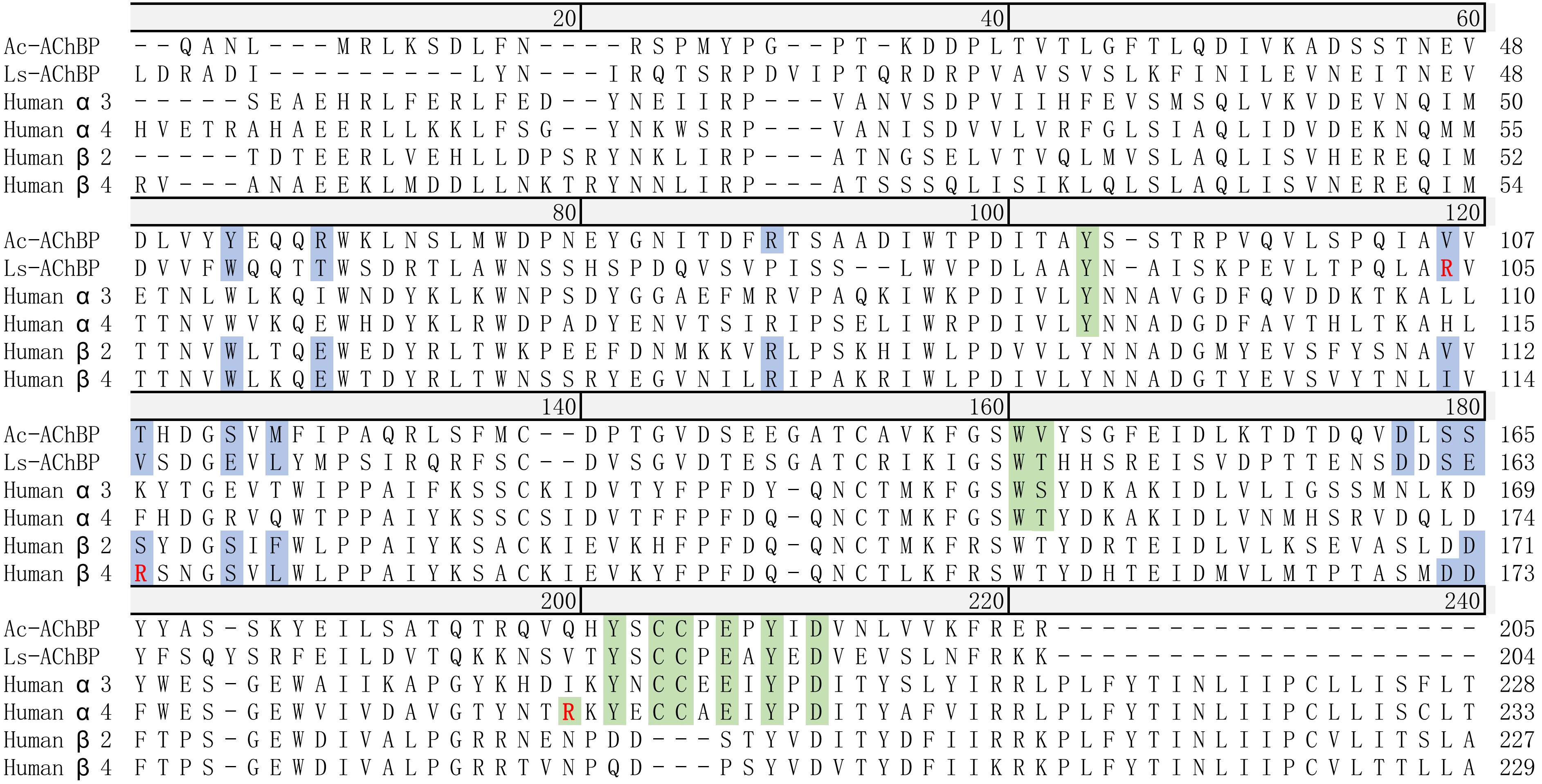

Figure S3 | Sequence alignment of Ac-AChBP, Ls-AChBP, and human α3, human α4, human β2, human β4 of nAChR subunits. Each subunit sequence numbering is shown at right side. Residues of the principal binding side that interact with α-conotoxin GIC are shown in light green; residues of the complementary binding side are in light blue. The Arg residues which could be a potential reason of decreased GIC affinity for the Ls-AChBP, human α4β2 nAChR and α3β4 nAChR are typed in red.
